# Supplementary material for: Fusion of Complement Fragment C3d Enhances Germinal Center Responses to HIV-1 Envelope Glycoproteins
Source: bioRxiv. 2025 Jul 1:2025.06.28.661730. Preprint. [Version 1] doi: 10.1101/2025.06.28.661730 (PMC12236797; doi:10.1101/2025.06.28.661730)
Supplement: 1 [file NIHPP2025.06.28.661730v1-supplement-1.pdf]

bioRxiv preprint doi: https://doi.org/10.1101/2025.06.28.661730; this version posted July 1, 2025. The copyright holder for this preprint (which was not certified by peer review) is the author/funder, who has granted bioRxiv a license to display the preprint in perpetuity. It is made available under aCC-BY 4.0 International license.

Supplementary Information:

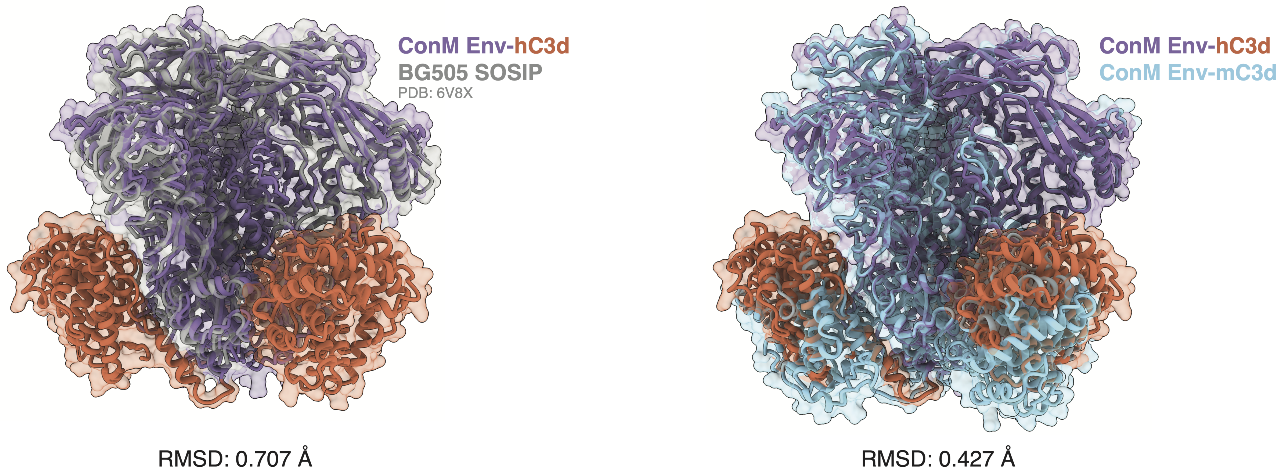

Figure S1: Structural comparison of ConM Env-hC3d with BG505 SOSIP and ConM Env-mC3d. Comparison of predicted ConM Env-hC3d with BG505 SOSIP (left) and ConM Env-murine C3d (right). BG505 SOSIP (PDB: 6V8X, left panel) and AlphaFold3-predicted ConM Env-mC3d show a high degree of similarity with the predicted ConM Env-hC3d structure. Root mean square deviation (RMSD) values are a measure of the average distance between atoms of superimposed structures, with lower values indicating a better fit.

bioRxiv preprint doi: https://doi.org/10.1101/2025.06.28.661730; this version posted July 1, 2025. The copyright holder for this preprint (which was not certified by peer review) is the author/funder, who has granted bioRxiv a license to display the preprint in perpetuity. It is made available under aCC-BY 4.0 International license.

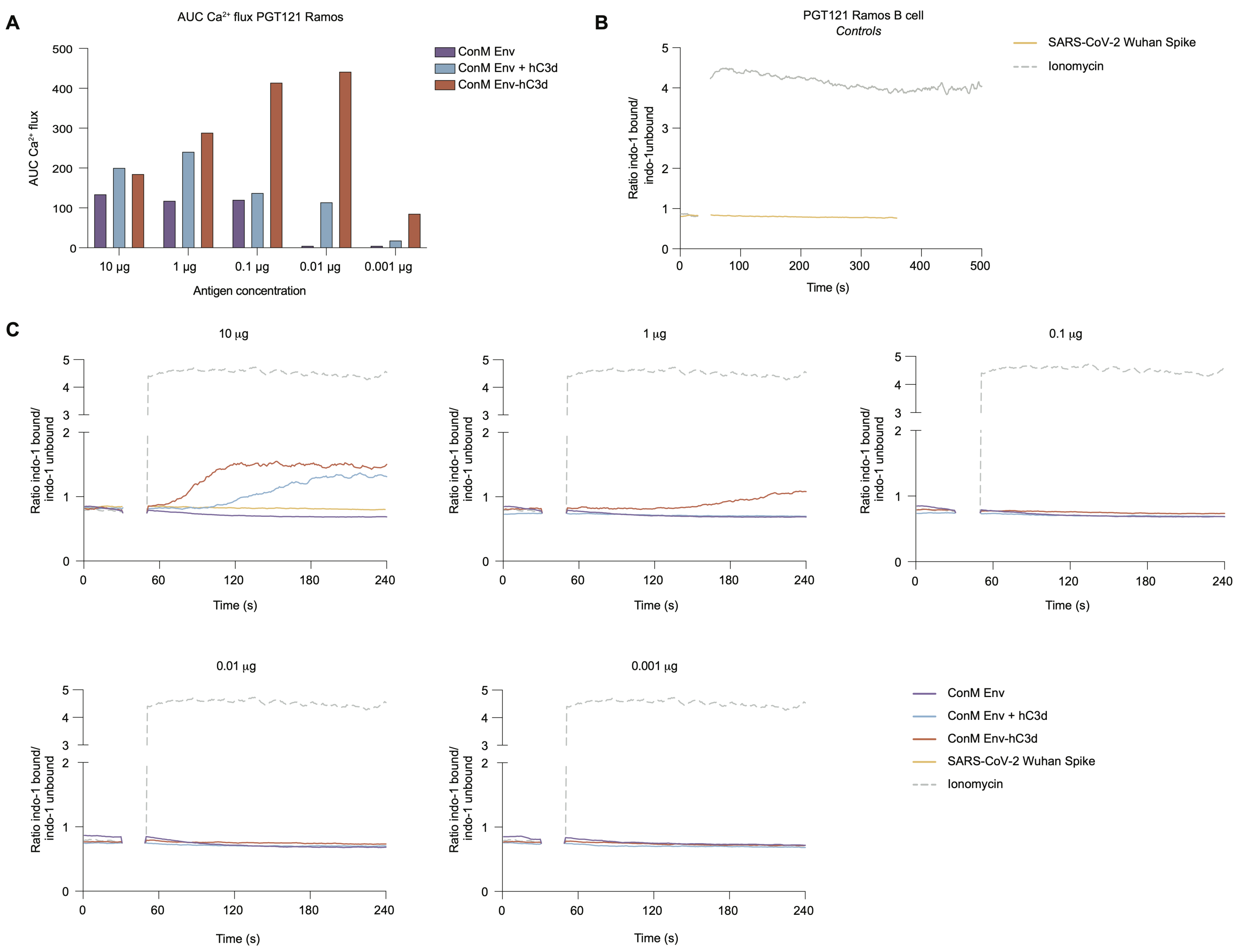

Figure S2: Activation of B cells expressing bNAb PGT121 or PGDM1400 by ConM Env and ConM Env-hC3d. (A) Area under the curve (AUC; 50–480 seconds) from calcium (Ca^{2+}) flux assays shown in Figure 2. PGT121-expressing Ramos B cells were stimulated with ConM Env, ConM Env-hC3d, or ConM Env supplemented with soluble hC3d (ConM Env + hC3d) at indicated concentrations. **(B)** Ca^{2+} flux assay with PGT121-expressing Ramos B cells stimulated with 1 µg/mL ionomycin serving as a positive control to establish maximal response and 10 µg/mL of SARS-CoV-2 Wuhan Spike protein as a negative control. **(C)** Activation of PGDM1400-expressing Ramos B cell assessed through a Ca^{2+} flux assay using equimolar amounts of ConM Env, ConM Env-hC3d, or ConM Env supplemented with recombinant hC3d (ConM Env + hC3d) at concentrations of 10 µg, 1 µg, 0.1 µg, 0.01 µg, and 0.001 µg (Env-equivalent mass). ConM Env and recombinant hC3d were co-administered at a molar ratio matching the stoichiometry of ConM Env-hC3d. Ionomycin was used at 1 µg/mL as positive control and 10 µg/mL of SARS-CoV-2 Wuhan Spike as negative control. A baseline without antigen was established between 0 and 30 s, after which the measurement was interrupted to add the antigen to the B cells (30–50 s).

bioRxiv preprint doi: https://doi.org/10.1101/2025.06.28.661730; this version posted July 1, 2025. The copyright holder for this preprint (which was not certified by peer review) is the author/funder, who has granted bioRxiv a license to display the preprint in perpetuity. It is made available under aCC-BY 4.0 International license.

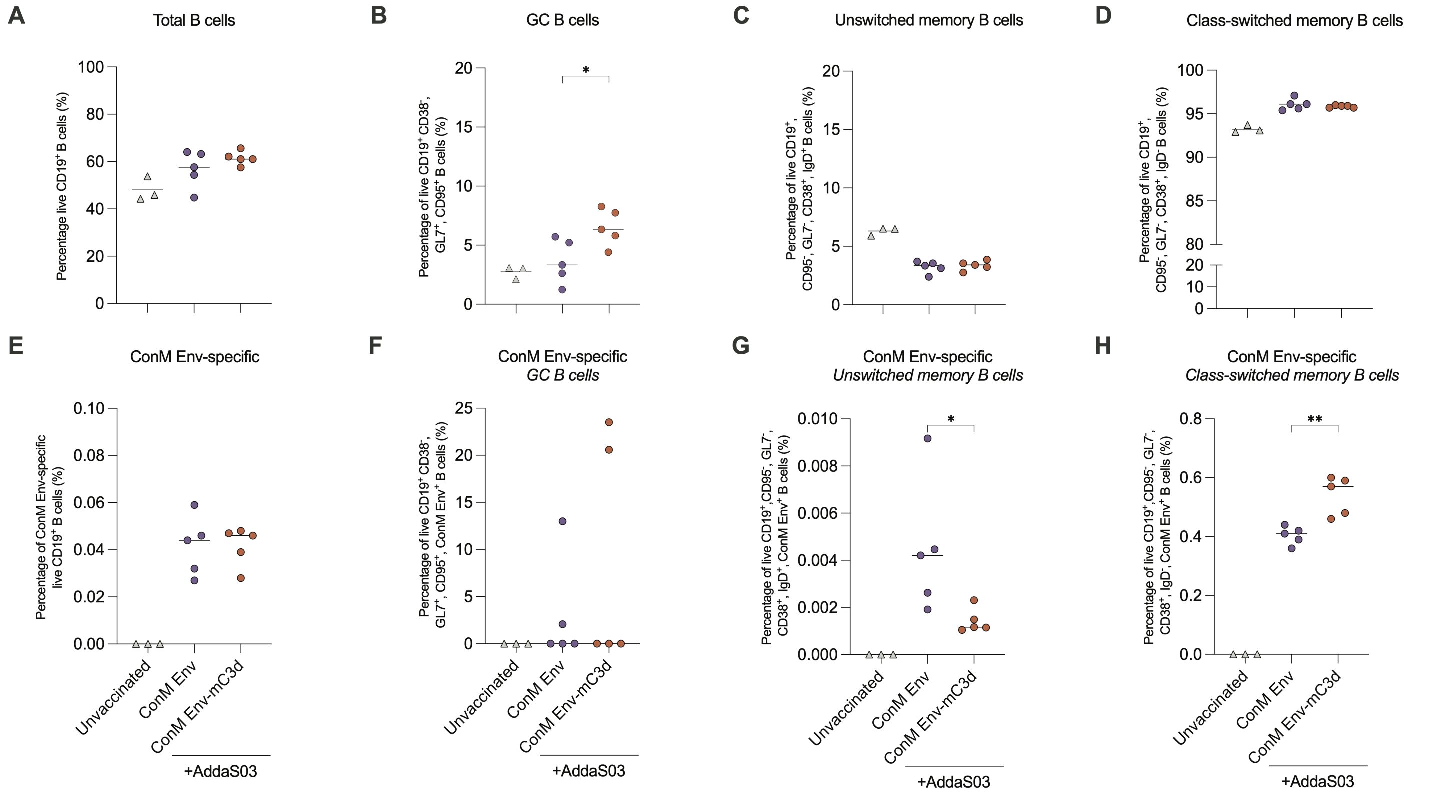

Figure S3: Splenic B cell populations following ConM Env or ConM Env-mC3d immunization. Mice were immunized as described in Figure 4A. Male mice (n = 5 per group) received three doses of either ConM Env or ConM Env-mC3d, each adjuvanted with AddaS03. An unvaccinated male control group (n = 3) was included for comparison. Splenocytes were harvested 4 weeks after the final immunization and analyzed by flow cytometry. **(A)** Frequency of total CD19$^+$ B cells. **(B)** Frequency of ConM Env-specific B cells. **(C)** Frequency of GC B cells (CD19$^+$ CD38$^-$ GL7$^+$ CD95$^+$). **(D)** Frequency of ConM Env-specific GC B cells. **(E)** Frequency of unswitched memory B cells (CD19$^+$ CD38$^+$ GL7$^-$ CD95$^-$ IgD$^+$). **(F)** Frequency of ConM Env-specific unswitched memory B cells. **(G)** Frequency of class-switched memory B cells (CD19$^+$ CD38$^+$ GL7$^-$ CD95$^-$ IgD$^-$). **(H)** Frequency of ConM Env-specific class-switched memory B cells. Data are shown as scatter dot plots with the median indicated by a horizontal line. Each dot represents one mouse. Statistical comparisons between groups were performed using two-tailed Mann–Whitney U tests. Significance is indicated as: ns ($p > 0.05$); * ($p < 0.05$); ** ($p < 0.01$); *** ($p < 0.001$).

bioRxiv preprint doi: https://doi.org/10.1101/2025.06.28.661730; this version posted July 1, 2025. The copyright holder for this preprint (which was not certified by peer review) is the author/funder, who has granted bioRxiv a license to display the preprint in perpetuity. It is made available under aCC-BY 4.0 International license.

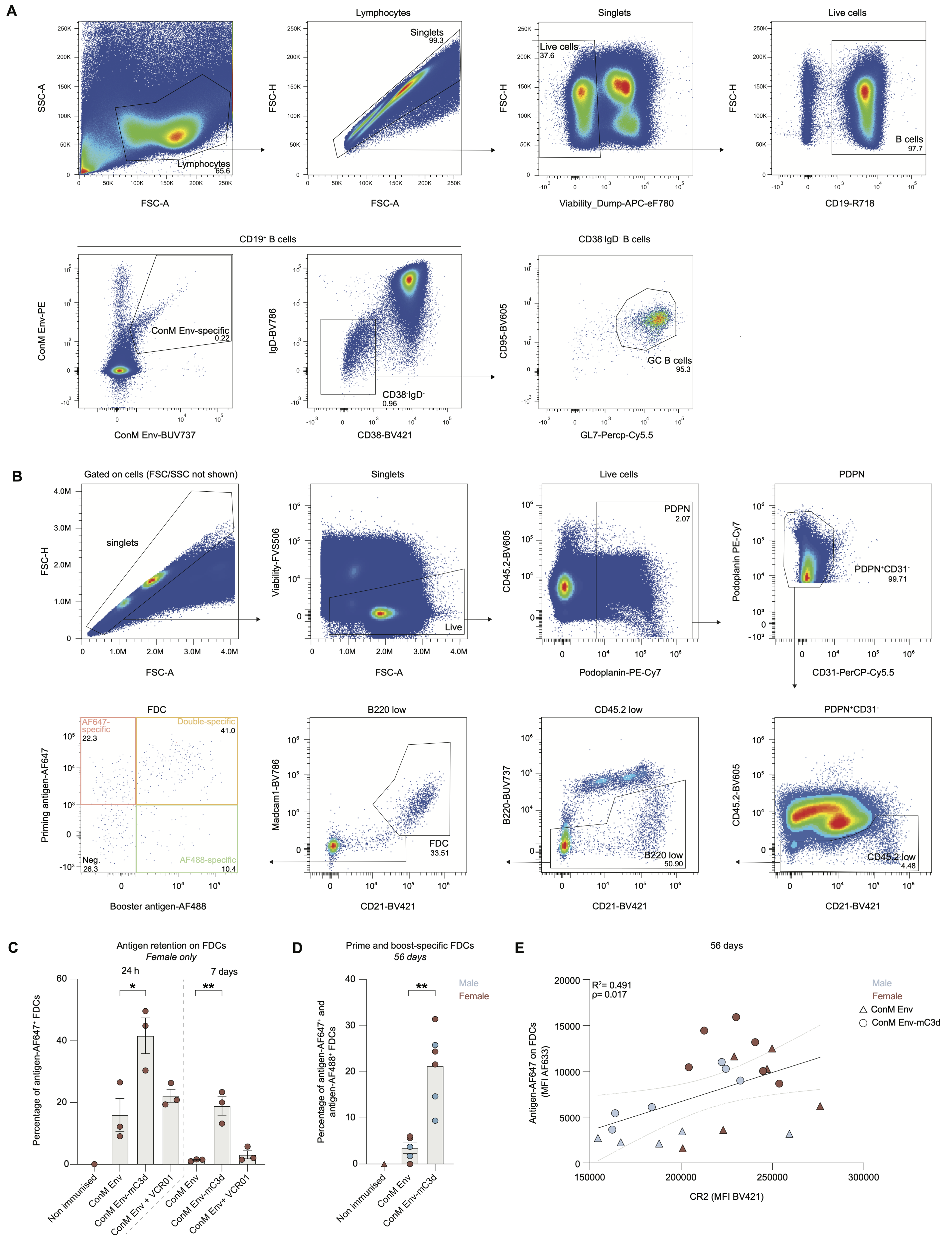

bioRxiv preprint doi: https://doi.org/10.1101/2025.06.28.661730; this version posted July 1, 2025. The copyright holder for this preprint (which was not certified by peer review) is the author/funder, who has granted bioRxiv a license to display the preprint in perpetuity. It is made available under aCC-BY 4.0 International license.

Figure S4: Flow cytometry gating, antigen retention, and correlation with CR2 expression in lymph node FDCs.(A) Gating strategy for identifying germinal center (GC) B cells and double-specific ConM Env^+ B cells in mouse lymph nodes. First, lymphocytes were selected, followed by exclusion of doublets (FSC-H vs. FSC-A) and dead cells (viability dye). B cells were identified as $CD19^+$, after which ConM Env-specific cells were gated as double-positive for BUV737- and PE-labeled ConM probes. GC B cells were defined within the $CD19^+$ population as $CD38^-$ IgD^- $GL7^+$ $CD95^+$. **(B)** Gating strategy for selecting FDCs from mouse lymph nodes involved excluding hematopoietic ($CD45^+$ $PDPN^-$) and B cells ($B220^+$), while selecting stromal cells ($PDPN^+$), non-endothelial ($CD31^-$), that express high levels of CR2 ($CD21/35^{hi}$) and integrin receptor $Madcam1^+$. **(C)** Bar graph depicting the percentage of antigen-$AF647^+$ FDCs ($CD45^-$,$B220^-$,$CD31^-$,$PDPN^+$,$CD21/35^{hi}$,$Madcam1^+$) at 24 hours and 7 days post-immunization in female mice treated with ConM Env-AF647, ConM Env-mC3d-AF647, and ConM Env-AF647 + VRC01, as described in Figure 5A. **(D)** Percentage of double-specific antigen-$AF647^+$ FCDs (prime-specific) and boost antigen-$AF488^+$ FDCs (boost-specific) for mice treated as described in Figure 5D, assessed at 56 days post-immunization. Sex differentiation is shown with females in maroon and males in light blue. Data are shown as bar plots with mean ± SEM. Each dot represents one mouse. Statistical comparisons were performed using two-tailed Mann–Whitney U tests. Significance is indicated as: ns ($p > 0.05$); * ($p < 0.05$); ** ($p < 0.01$). **(E)** Correlation between CR2 expression and antigen retention on FDCs in draining lymph nodes at 56 days post-immunization. Data from all mice were pooled, and a Pearson correlation was used to assess the relationship between CR2 expression (measured as CD21-BV421 MFI) and antigen retention on FDCs (measured as antigen-AF647 MFI); the R^2 and p values shown reflect this analysis. Each symbol represents an individual mouse: circles indicate ConM Env-mC3d-immunized mice, and triangles indicate ConM Env-immunized mice. Blue symbols represent males, and maroon symbols represent females. The solid line indicates the best-fit linear regression; shaded dotted lines show the 95% confidence interval.
